# The Efficacy of Pyrotinib as a Third- or Higher-Line Treatment in HER2-Positive Metastatic Breast Cancer Patients Exposed to Lapatinib Compared to Lapatinib-Naive Patients: A Real-World Study

**DOI:** 10.3389/fphar.2021.682568

**Published:** 2021-08-26

**Authors:** D. J Ouyang, Q. T Chen, M. Anwar, N. Xie, Q. C. Ouyang, P. Z. Fan, L. Y. Qian, G. N. Chen, E. X. Zhou, L. Guo, X. W. Gu, B. N. Ding, X. H. Yang, L. P. Liu, C. Deng, Z. Xiao, J. Li, Y. Q. Wang, S. Zeng, Shouman Wang, Wenjun Yi

**Affiliations:** ^1^ Department of General Surgery, The Second Xiangya Hospital, Central South University, Changsha, China; ^2^ Department of General Surgery, Xiangya Hospital, Central South University, Changsha, China; ^3^ Department of Internal Medicine of Breast, The Affiliated Cancer Hospital of Xiangya School of Medicine, Central South University, Changsha, China; ^4^ Department of Breast and Thyroid Surgery, Hunan Provincial People’s Hospital, Changsha, China; ^5^ Department of Breast and Thyroid Surgery, Third Xiangya Hospital, Central South University, Changsha, China; ^6^ Department of Breast Surgery, Xiangya Hospital, Central South University, Changsha, China; ^7^ Department of Oncology, The Affiliated Cancer Hospital of Xiangya School of Medicine, Central South University, Changsha, China; ^8^ Department of Oncology, The Second Xiangya Hospital, Central South University, Changsha, China; ^9^ Department of Traditional Chinese Medicine, The Affiliated Cancer Hospital of Xiangya School of Medicine, Central South University, Changsha, China; ^10^ Department of Internal Medicine–Oncology, Xiangya Hospital, Central South University, Changsha, China

**Keywords:** pyrotinib, lapatinib-treated, lapatinib-naive, HER2 breast cancer, metastases

## Abstract

**Background:** Pyrotinib is a novel irreversible pan-ErbB receptor tyrosine kinase inhibitor. Evidence of the efficacy of pyrotinib-based treatments for HER2-positive metastatic breast cancer (MBC) in patients exposed to lapatinib is limited.

**Methods:** Ninety-four patients who received pyrotinib as a third- or higher-line treatment for HER2-positive MBC were included in this retrospective study. The primary and secondary endpoints were overall survival (OS) and progression‐free survival (PFS). Propensity score matching (PSM) and inverse probability of treatment weighting (IPTW) analysis were implemented to balance important patient characteristics between groups.

**Results:** Thirty (31.9%) patients were pretreated with lapatinib and subsequently received pyrotinib as an anti-HER2 treatment, and 64 (68.1%) patients did not receive this treatment. The OS and PFS indicated a beneficial trend in lapatinib-naive group compared to lapatinib-treated group in either the original cohort (PFS: 9.02 vs 6.36 months, *p* = 0.05; OS: 20.73 vs 14.35 months, *p* = 0.08) or the PSM (PFS: 9.02 vs 6.08 months, *p* = 0.07; OS: 19.07 vs 18.00 months, *p* = 0.61) or IPTW (PFS: 9.90 vs 6.17 months, *p* = 0.05; OS: 19.53 vs 15.10 months, *p* = 0.08) cohorts. Subgroup analyses demonstrated lapatinib treatment-related differences in PFS in the premenopausal subgroup and the no prior trastuzumab treatment subgroup, but no significant differences were observed in OS.

**Conclusion:** Pyrotinib-based therapy demonstrated promising effects in HER2-positive MBC patients in a real-world study, especially in lapatinib-naive patients, and also some activity in lapatinib-treated patients.

## Introduction

Among patients with metastatic breast cancer (MBC), more than 20% have HER2-positive disease (Cobleigh et al., 2020; Howlader et al., 2014). Although this subtype of breast cancer has been historically associated with poor outcomes, the development of anti-HER2-targeted therapies has notably increased the median progression-free survival (PFS) and overall survival (OS) of patients (Slamon et al., 2001; Dawood et al., 2010; Swain et al., 2013; Mendes et al., 2015; Swain et al., 2015; Jain et al., 2018; Tripathy et al., 2020). Currently, tyrosine kinase inhibitors (TKIs) are officially approved by the International and Chinese Food and Drug Administrations for HER2-positive recurrence and MBC as second- or higher-line treatments (Ryan et al., 2008; Deeks, 2017; Blair, 2018).

Four TKIs are used to treat HER2-positive MBC, namely, lapatinib, tucatinib, neratinib, and pyrotinib (Xuhong et al., 2019; Lee, 2020). All of them were pan-ErbB receptors TKIs except tucatinib, which was a single HER2-targeted TKI (Wong et al., 2009; Awada et al., 2016; Li et al., 2017; Lin et al., 2020). Clinical trial results and our previous real-world study indicated that pyrotinib plus capecitabine had significantly superior efficacy and resulted in greater PFS than lapatinib combined with capecitabine (Jiang et al., 2019; Ma et al., 2019; Chen et al., 2020; Xuhong et al., 2020; Xu et al., 2021). Pyrotinib also demonstrated promising effects in brain metastatic HER2-positive breast cancer regardless of whether patients were previously treated with trastuzumab (Ma et al., 2019; (Anwar et al., 2021). The TBCRC022 study indicated that neratinib plus capecitabine was effective in HER2-positive patients with brain metastasis of breast cancer among the lapatinib-treated group (Freedman et al., 2019). However, whether pyrotinib is effective in patients after lapatinib treatment remains controversial (Lin et al., 2020; Song et al., 2020). This study was conducted after obtaining our final follow-up data to evaluate the effectiveness of pyrotinib as a third- or higher-line treatment. The aim of the study is to report the results of pyrotinib therapy in patients with and without prior lapatinib exposure before and after propensity score matching (PSM) analysis and inverse probability of treatment weighting (IPTW) analysis, with the hope of providing evidence of the effectiveness of pyrotinib-based treatment after failure of lapatinib-treated therapy.

## Methods

### Patient Eligibility and Data Collection

**FIGURE 1:**
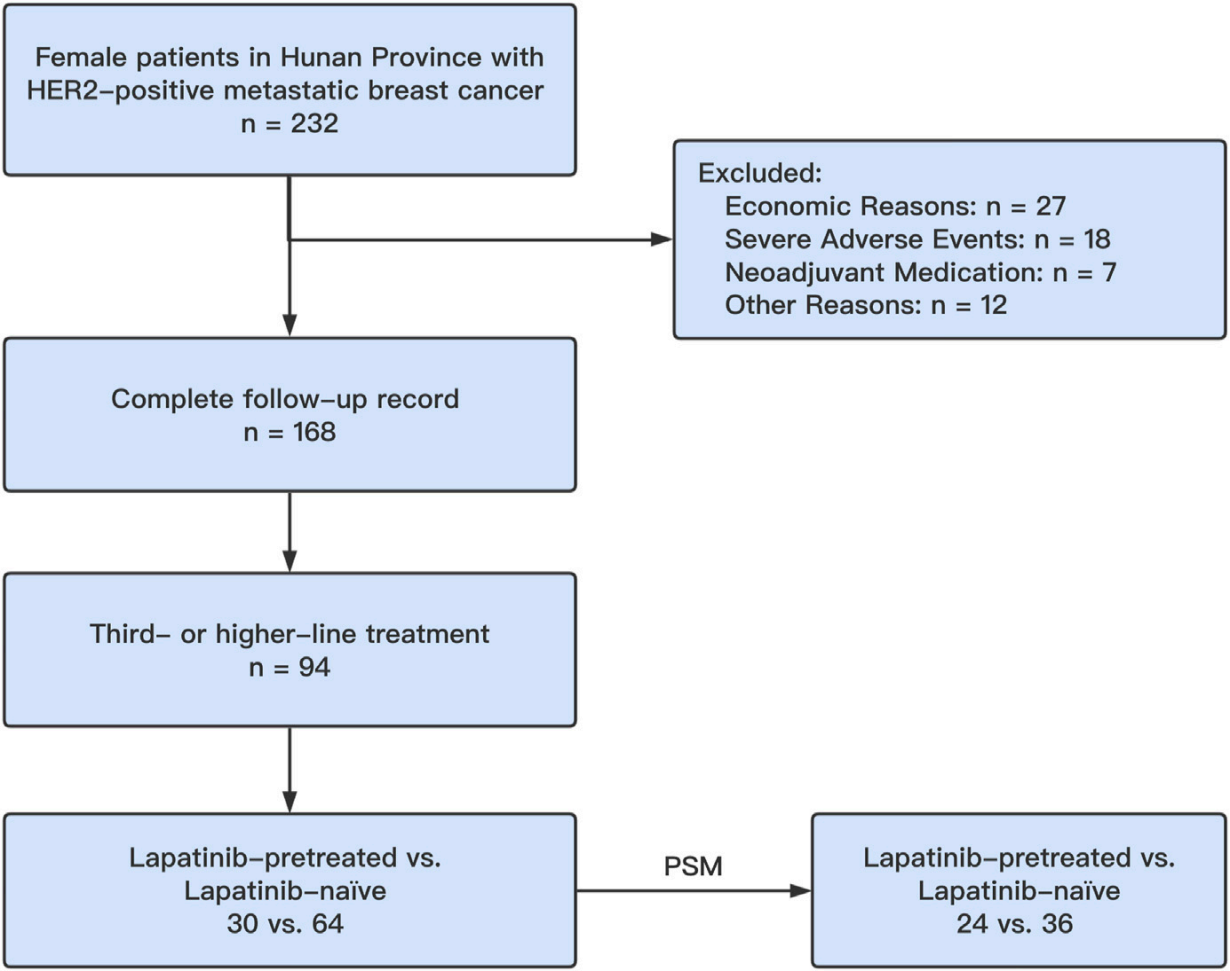
CONSORT diagram for patient selection for the study. PSM, propensity score matching.

One hundred sixty-eight female patients with HER2-positive MBC were enrolled from June 2018 to August 2019. The follow-up period of the present study lasted until December 2020. Among these patients, 94 were treated with pyrotinib as a third- or higher-line treatment. Thirty (31.9%) patients had previously been treated with lapatinib and subsequently received pyrotinib-based therapy, and 64 (68.1%) patients had not been treated with lapatinib in this retrospective, multicenter, real-world study. Using PSM, a total of 60 patients (24 lapatinib-treated patients (40.0%) versus 36 lapatinib-naive patients (60.0%)) were matched, and the two groups were confirmed to have similar baseline clinical data (*p* > 0.05). Pyrotinib treatment was identical to that in our previous study (Geyer et al., 2006; Cameron et al., 2010; Xu et al., 2011) (Figure 1). The inclusion criteria were as follows (Cobleigh et al., 2020): confirmed HER2 positivity by immunohistochemistry (IHC) or fluorescence *in situ* hybridization (FISH) according to the HER2 status testing guidelines (Wolff et al., 2018; Howlader et al., 2014) stable vital signs and adequate physiological function (heart, liver, and kidney); and (Tripathy et al., 2020) a measurable lesion. The exclusion criteria were as follows (Cobleigh et al., 2020): discontinued pyrotinib treatment (Howlader et al., 2014); pyrotinib use in a neoadjuvant therapy setting (Tripathy et al., 2020); severe adverse side effects could not be controlled by dose reduction or adjuvant medication; and (Dawood et al., 2010) dropped out for other unknown reasons.

The pyrotinib treatment stage was defined as follows: first-line treatment was defined as the treatment of a patient with *de novo* stage IV breast cancer who was not treated previously with anti-HER2 medications or treatment of a patient with recurrence >12 months after discontinuation of trastuzumab. Second-line treatment was administered to patients with recurrence within 12 months of discontinuation of trastuzumab, recurrence during adjuvant therapy with trastuzumab, or progression following first-line treatment. Third- or higher-line treatment was administered to patients with progression or recurrence following second-line treatment and for whom any one of the anti-HER2 or chemotherapeutic drugs had been changed.

All patients and/or their immediate families understood and consented to participate in this study and provided written informed consent for clinical data access, scheduled follow-up, and survival analysis. The Ethics Committee of the Second Xiangya Hospital of Central South University reviewed and approved the study.

### Endpoint Definition and Assessments

OS, the primary endpoint of our study, was defined as the time from enrollment until death due to any cause or the latest date the patient was known to be alive. The secondary endpoint, PFS, was defined as the time from drug administration to death or disease progression (whichever occurred first). For patients without OS/PFS events, the follow-up information was estimated by each center’s staff based on the Response Evaluation Criteria in Solid Tumours (RECIST) v1.1 criterion. Each patient underwent a 14- to 21-day clinical follow-up schedule and 2 to 3 drug cycles (6–9 weeks) of imaging follow-up (computed tomography (CT), ultrasound, magnetic resonance imaging (MRI), positron emission tomography (PET) scan, PET/CT scan, and bone scan) after the beginning of pyrotinib treatment until the primary endpoint was reached.

### Propensity Score Matching and Inverse Probability of Treatment Weighting

The critical covariate (metastatic site) exhibited heterogeneity between the lapatinib-treated and lapatinib-naive groups (Table 1), possibly affecting the outcomes from a clinical perspective. To balance the heterogeneous characteristics between the two groups, we implemented PSM using the R package “MatchIt” version 4.1.0 with the following settings: 1:2 pairing, nearest-neighbor methods, and a caliper of 0.02 (Pattanayak et al., 2011). After PSM, all categories were comparable (Table 1). Inverse probability of treatment weighting-adjusted (IPTW-adjusted) survival analysis was applied to reduce the differences in baseline variables.

**TABLE 1:** Characteristics of patients who received pyrotinib as third- or higher-line therapy who previously were or were not treated with lapatinib.

Category	Before PSM			After PSM		
	Lapatinib-naive	Lapatinib-treated	*p*-Value	Lapatinib-naive	Lapatinib-treated	*p*-Value
	No. (%)	No. (%)		No. (%)	No. (%)	
**Age**						
<50	31 (48.4)	18 (60.0)	0.377	17 (47.2)	15 (62.5)	0.369
≥50	33 (51.6)	12 (40.0)		19 (52.8)	9 (37.5)	
**ECOG Scale**
0–1	63 (98.4)	27 (90.0)	0.181	35 (97.2)	23 (95.8)	1.000
≥2	1 (1.6)	3 (10.0)		1 (2.8)	1 (4.2)	
**Menopausal Status**						
Postmenopausal	30 (46.9)	17 (56.7)	0.507	17 (47.2)	13 (54.2)	0.792
Premenopausal	34 (53.1)	13 (43.3)		19 (52.8)	11 (45.8)	
**HR Status**
Positive	37 (57.8)	16 (53.3)	0.853	20 (55.6)	13 (54.2)	1.000
Negative	27 (42.2)	14 (46.7)		16 (44.4)	11 (45.8)	
**Prior trastuzumab treatment**						
No prior trastuzumab	19 (29.7)	5 (16.7)	0.273	12 (33.3)	4 (16.7)	0.258
Previous use of trastuzumab	45 (70.3)	25 (83.3)		24 (66.7)	20 (83.3)	
**Treatment type**
Pyrotinib + Capecitabine	45 (70.3)	16 (53.3)	0.102	24 (66.7)	12 (50.0)	0.330
Pyrotinib + Abraxane	12 (18.8)	5 (16.7)		8 (22.2)	5 (20.8)	
Pyrotinib + Trastuzumab	2 (3.1)	1 (3.3)		1 (2.8)	1 (4.2)	
Pyrotinib + Others	5 (7.8)	8 (26.7)		3 (8.3)	6 (25.0)	
**Metastatic Site**						
Soft tissue and/or bone	9 (14.1)	5 (16.7)	<0.001	9 (25.0)	5 (20.8)	0.480
Lung and/or liver	44 (68.8)	8 (26.7)		16 (44.4)	8 (33.3)	
Brain and/or others	11 (17.2)	17 (56.7)		11 (30.6)	11 (45.8)	
**Total**	**64**	**30**		**36**		**24**

**Abbreviations:** PSM, propensity score matching; ECOG, Eastern Cooperative Oncology Group; HR, hormone receptor.

### Statistical Analyses

Pearson’s chi-squared test or Fisher’s exact test was utilized to assess the heterogeneity of categorical variables among the lapatinib-treated and lapatinib-naive groups. Survival curves for OS and PFS were constructed using the Kaplan–Meier methodology, and the distribution was estimated using the log-rank test. Median OS times and PFS were calculated and reported. Hazard ratios (HRs) and 95% confidence intervals (CIs) for OS and PFS were computed using a univariable Cox proportional hazards regression model (using the R package “survminer”) and are presented as Forrest plots (using the R package “forestplot”). Statistical analyses and data visualization were performed using R (https://www.r-project.org/version 4.0.3) and RStudio (R-Studio Inc., Boston, United States version 1.3.1056). A *p*-value of less than 0.05 indicated statistical significance.

## Results

### Baseline Characteristics

Of 94 eligible patients, the median age of the 94 patients was 48.5 years (range 28–71 years). Ninety (95.7%) patients had an Eastern Cooperative Oncology Group (ECOG) performance status of 0–1. Most patients were treated with pyrotinib plus capecitabine and had prior trastuzumab treatment. In the lapatinib-naive cohort, 44 (68.8%) patients were with lung and/or liver metastasis, 33 (51.6%) were ≥50 years old, and 34 (53.1%) had a premenopausal status. The hormone receptor status was similar between groups. The PSM cohort showed similar but more balanced patient characteristics than those in the initial cohort. The baseline clinical features of the patients before and after PSM are summarized in Table 1. The median PFS time of the patients was 7.54 months (95% CI 6.67–10.67 months), and the median OS time was 18.67 months (95% CI 14.97–24.47 months) (Supplementary Figure S1).

### Patient Outcomes After Changing Tyrosine Kinase Inhibitor Treatment

**FIGURE 2:**
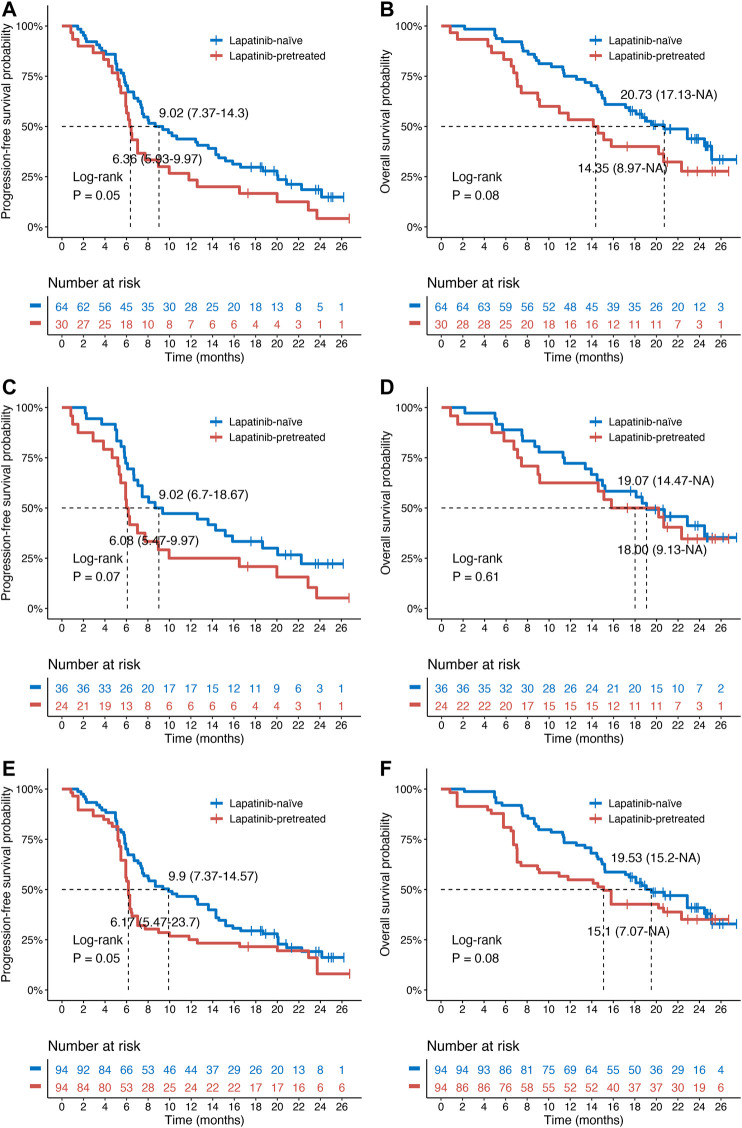
Kaplan–Meier survival curves for patients with HER2-positive MBC treated with pyrotinib as a third- or higher-line treatment. **(A,B)** Progression-free survival (PFS)/overall survival (OS) of lapatinib-naive (*n* = 64) and lapatinib-treated (*n* = 30) patients in the original cohort. **(C,D)** PFS/OS of lapatinib-naive (*n* = 36) and lapatinib-treated (*n* = 24) patients in the PSM cohort. **(E,F)** PFS/OS of lapatinib-naive (*n* = 94) and lapatinib-treated (*n* = 94) patients in the IPTW-adjusted cohort. The *p*-values were determined by univariate log-rank tests. PSM, propensity score matching; IPTW, inverse probability of treatment weighting.

The numbers of PFS events in the lapatinib-naive group were 51/64 (before PSM) and 27/36 (after PSM), and the numbers of OS events were 36/64 and 21/36, respectively. In the lapatinib-treated group, the numbers of PFS events were 28/30 (before PSM) and 22/24 (after PSM), and the numbers of OS events were 21/30 and 15/24, respectively. Kaplan–Meier survival curves for OS and PFS were constructed to compare the survival distribution according to previous lapatinib treatment (Figure 2). The PFS and OS of the lapatinib-naive group were 43.8 and 75.0% at 12 months and 29.7% and 57.8% at 18 months, respectively. Comparatively, the PFS and OS of the lapatinib-treated group were 23.3 and 53.3% at 12 months and 16.7% and 40.0% at 18 months, respectively. The log-rank test results indicated a beneficial trend in the lapatinib-naive group compared to the lapatinib-treated group in terms of PFS (9.02 (7.37–14.30) vs 6.36 (5.93–9.97) *p* = 0.05, Figure 2A) and OS (20.73 (17.13–NA) vs 14.35 (8.97–NA) *p* = 0.08, Figure 2B). We also confirmed this finding by performing analysis between the two groups in terms of PFS (9.02 (6.70–18.67) vs 6.08 (5.47–9.97) *p* = 0.07, Figure 2C) and OS (19.07 (14.47–NA) vs 18.00 (9.13–NA) *p* = 0.61, Figure 2D) in the PSM cohort and in IPTW-adjusted cohort (PFS: 9.90 (7.37–14.57) vs 6.17 (5.47–23.70) *p* = 0.05, Figure 2E, and OS: 19.53 (15.20–NA) vs 15.10 (7.07–NA) *p* = 0.08, Figure 2F).

In the lapatinib-naive cohort, 24 patients (37.5%) achieved a partial response (PR), and two patients (3.10%) achieved a complete response (CR), resulting in an objective response rate (ORR) of 40.60%. In the lapatinib-treated cohort, 11 patients (36.70%) achieved a PR, and one patient (3.30%) achieved a CR, resulting in an ORR of 40.00%.

### Subgroup Analysis

**FIGURE 3:**
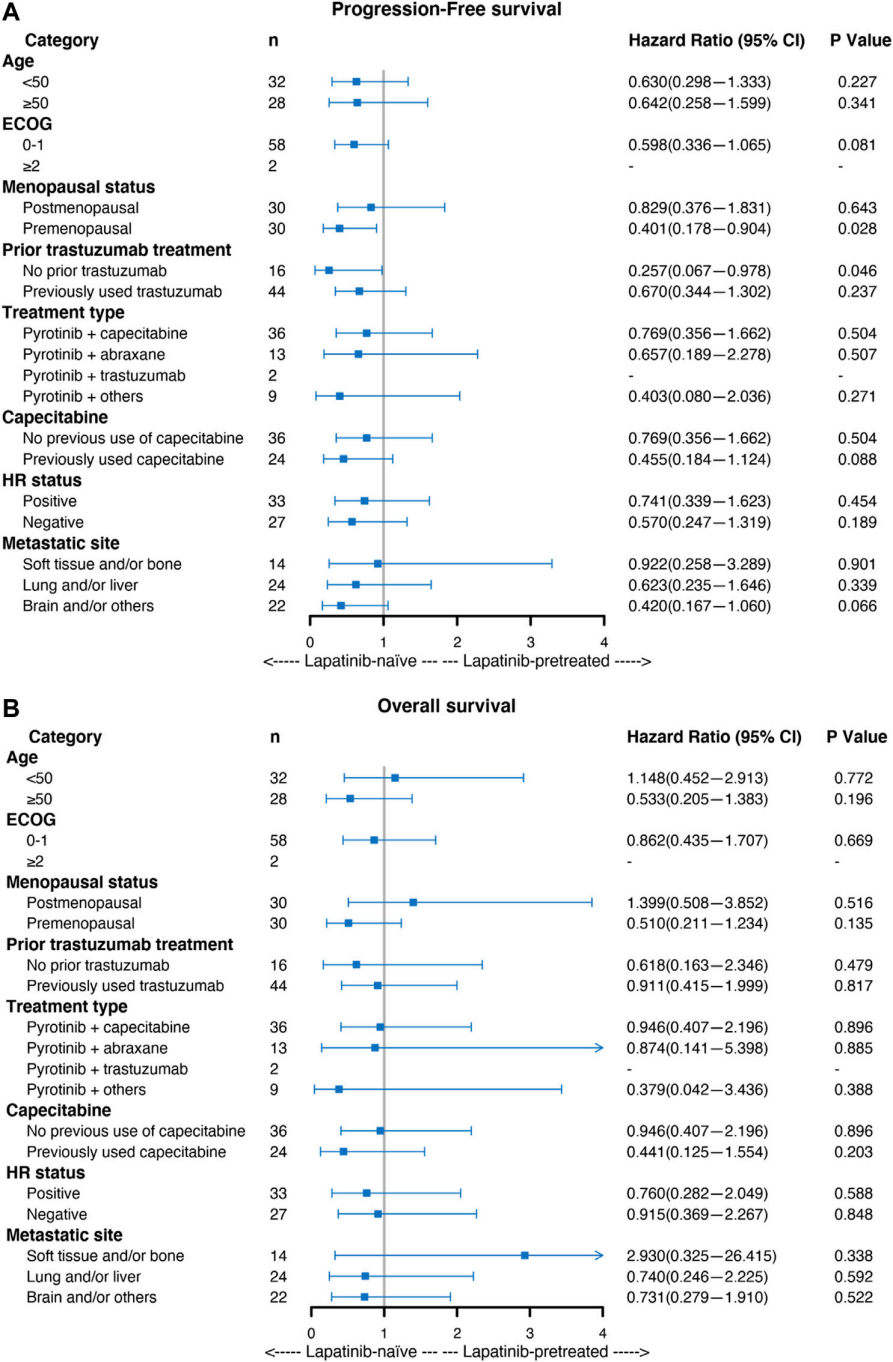
Forest plot of the subgroup analysis of patients with HER2-positive MBC with regard to **(A)** progression-free survival (PFS) and **(B)** overall survival (OS). The hazard ratio (HR) and 95% confidence interval (CI) and the *p*-values were determined by Cox proportional hazard regression.

A subgroup analysis was performed to investigate the effect of previous lapatinib treatment on PFS and OS. Forest plots of the subgroup analysis are shown in Figure 3.

Univariable Cox analysis including the lapatinib-treated and lapatinib-naive groups showed similar outcomes. Most subgroups showed no significant difference in PFS (Figure 3A), except for the premenopausal subgroup (HR = 0.401, 95% CI 0.178–0.904, *p* = 0.028) and the subgroup without previous use of trastuzumab (HR = 0.257, 95% CI 0.067–0.978, *p* = 0.046). Similarly, no significant differences were found in OS in any subgroup analyses (Figure 3B).

## Discussion

HER2-positive MBC has a poor prognosis and a short survival time (4). Only 13.2% of patients survive for more than 5 years if they do not receive treatments that target HER2(4). Conversely, the continuous development and widespread use of anti-HER2 drugs such as trastuzumab (Slamon et al., 2001; Burstein et al., 2007; Robert et al., 2006), pertuzumab (Baselga et al., 2012), TDM1 (Verma et al., 2012), and lapatinib (Geyer et al., 2006; Cameron et al., 2010; Xu et al., 2011) have significantly prolonged the median survival time of HER2-positive MBC patients. Moreover, China has recently authorized the use of pyrotinib for HER2-positive MBC patients.

Lapatinib and pyrotinib are both small molecule TKIs. Lapatinib reversibly inhibits HER1 and HER2, while pyrotinib inhibits HER1, HER2, and HER4 (Ma et al., 2017; Li et al., 2019). The curative effect of pyrotinib is stronger than that of lapatinib because of the conjugated double bond structure (Ma et al., 2017; Li et al., 2019). Previous randomized controlled trials on pyrotinib have not included lapatinib-treated patients, resulting in a lack of evidence to guide practice for follow-up treatment after lapatinib failure. In this study, 24 patients were considered to be lapatinib-treated after PSM analysis, which resulted in an ORR of 40.0% and median PFS of 6.08 months. Furthermore, IPTW analysis showed 6.17 months of PFS in patients who were exposed to lapatinib previously. We compared our results with those of two other real-world studies. Lin et al. (2020) and Song et al. (2020) reported median PFS times of 5.4 months (ORR 23.2%) and 7.9 months (ORR 22.2%), respectively. Our results showed a median PFS of 9.02 months (PSM analysis) and 9.90 months (IPTW analysis) in the lapatinib-naive group, which was better than that from Lin’s study (9.0 months) and Song’s study (7.2 months). The differences between the studies of Lin and Song may be due to selection bias. To minimize this bias, our study assessed the efficacy of pyrotinib by applying a PSM and IPTW approach. Additionally, our results first revealed the OS of pyrotinib-based therapy, with survival times of 19.07 and 18.00 months (PSM analysis) and 19.53 and 15.10 months (IPTW analysis) for the lapatinib-naive and lapatinib-treated groups, respectively. Therefore, our study suggested that pyrotinib is still effective in patients who have lapatinib treatment failure.

Another TKI neratinib also showed good therapeutic effects (Geyer et al., 2006; Cameron et al., 2010; Xu et al., 2011). The NALA study reported median PFS times of 8.8 months in the neratinib plus capecitabine group and 6.6 months in the lapatinib plus capecitabine group (Geyer et al., 2006; Cameron et al., 2010; Xu et al., 2011), and the NEfERT-T trial reported a PFS time of 12.9 months in the neratinib plus paclitaxel group (Geyer et al., 2006; Cameron et al., 2010; Xu et al., 2011), suggesting that the curative effect of neratinib is stronger than that of lapatinib. Among patients treated with neratinib, the PFS time was 3.1 months for those previously treated with lapatinib, and the PFS time was and 5.5 months in the lapatinib-naive cohort (Freedman et al., 2019). Thus, this finding indicated that the therapeutic effect of pyrotinib in lapatinib-naive patients has a similar beneficial trend to that of neratinib. Therefore, giving priority to pyrotinib treatment may increase survival benefits, but more detailed clinical studies are needed in the future.

Our study was retrospective, and thus, the groups could not be prospectively randomized; therefore, it was subject to limitations, including a lack of some clinical factors, such as combined treatment, and possible selection bias. The sample size should be further expanded in clinical randomized controlled studies.

In conclusion, pyrotinib-based therapy exhibited potential effects on HER2-positive MBC patients in a real-world study, regardless of whether lapatinib treatment was previously administered or not. Particularly for patients without lapatinib exposure, they seemed to benefit more from pyrotinib-based therapy, reaching a better prognosis, which still awaits more solidate verification.

## Data Availability

The code and datasets analyzed during the present study are available from the corresponding authors upon reasonable request.
