# Supplementary material for: The Efficacy of Pyrotinib as a Third- or Higher-Line Treatment in HER2-Positive Metastatic Breast Cancer Patients Exposed to Lapatinib Compared to Lapatinib-Naive Patients: A Real-World Study
Source: Front Pharmacol. 2021 Aug 26;12:682568. doi: 10.3389/fphar.2021.682568 (PMC8428978; doi:10.3389/fphar.2021.682568)
Supplement: Supplementary file 1 [file Image1.pdf]

Supplementary Material

1 Supplementary Figures

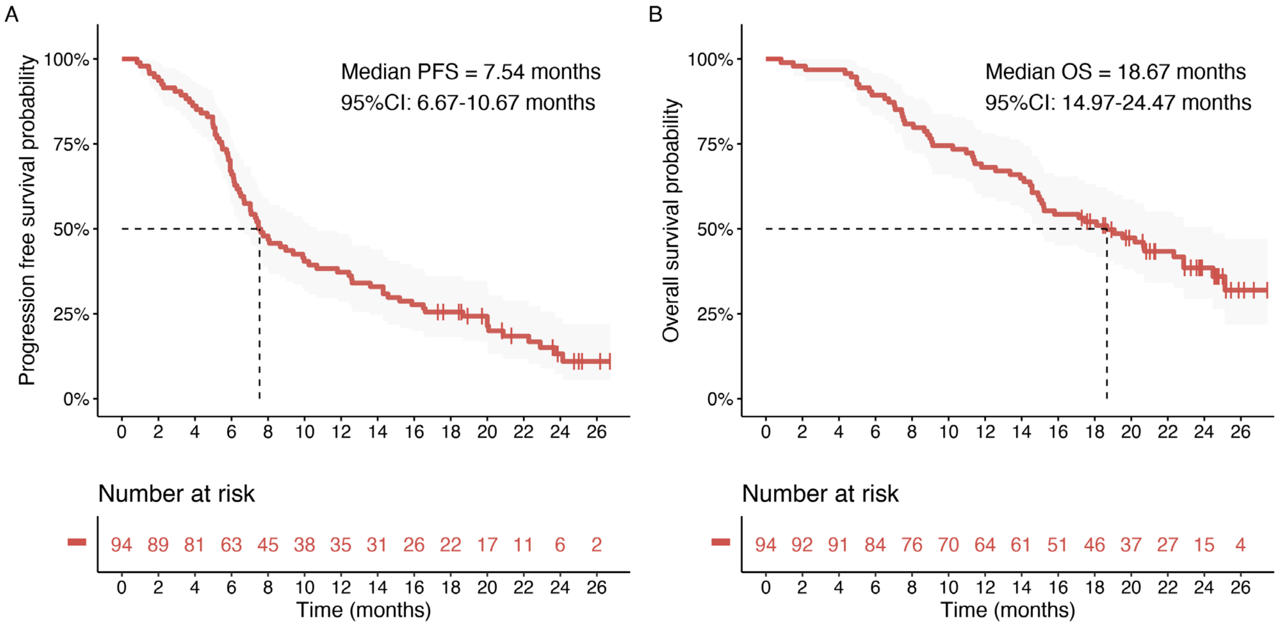

Supplementary Figure 1. Kaplan–Meier survival curves of PFS and OS for the entire cohort of patients with HER2-positive MBC (n = 94).
